# Feeding value of sorghum stover fed to tropical hair sheep as complete rations in chop, mash, pellet, and block forms

**DOI:** 10.14202/vetworld.2021.2273-2281

**Published:** 2021-08-30

**Authors:** J. Raju, J. Narasimha, N. Nalini Kumari, T. Raghunanadan, V. Chinni Preetam, A. Ashok Kumar, P. Ravi Kanth Reddy

**Affiliations:** 1 Department of Animal Nutrition, PVNR Telangana Veterinary University, Hyderabad, Telangana, India; 2 Department of Plant Breeding, International Crops Research Institute for the Semi-arid Tropics, Hyderabad, Telangana, India; 3 Veterinary Dispensary, Department of Animal Husbandry, Taticherla, Prakasam, Andhra Pradesh, India

**Keywords:** block, crop residue, digestibility, feed processing, growth, lambs, mash, pellet

## Abstract

**Background and Aim:**

Poor palatability, low bulk density, and low nutritive value restrict the utilization of the crop residues as animal feeds. Altering the physical characteristics of feed by blending the roughage and concentrates in the form of complete feed improves the nutrient use efficiency and reduces the feed wastage, feed cost, and labor cost. The study aims to determine suitable processing methods (mash, pellet, or block forms) for efficient utilization of sorghum stover-based complete rations vis-a-vis conventional feeding methods in sheep.

**Materials and Methods:**

The sorghum stover was incorporated in complete rations with roughage to concentrate ratio of 50:50 proportion in a growth trial of 120 days. The feed ingredients were chaffed, ground in a hammer mill, passed through expander-extruder, and compressed by feed block machine to prepare chop, mash, pellet, and block form of rations, respectively. Twenty-four male intact growing Nellore×Deccani cross lambs (3.5±0.5 months age, 14.50±0.41 kg (mean ± SD) at the start of the experiment) were divided into four experimental groups of six animals each in a complete randomized design. The experimental rations were randomly allotted to each group and evaluated for their intake, nutrient utilization, and growth performance.

**Results:**

The sheep fed on pellet-based ration consumed a higher (p<0.05) quantity of dry matter. The digestibility coefficients of organic matter, crude protein, and neutral detergent fiber were higher (p<0.05) in processed rations (mash, pellet, or block). Further, the nitrogen balance (g/d) was higher (p<0.05) in the mash, pellet, and block form of rations, compared to chopped ration. The processing method did not influence calcium or phosphorous balance parameters, except for their urinary losses, which showed an increasing trend (p=0.07). The ram lambs fed with pelleted ration showed higher (p<0.05) weight gain than mash, block, or chaff forms. The efficiency of feed utilization in gaining one kg body weight was higher (p<0.05) in lambs fed a pelleted diet. Feeding pelletized ration was more economical to gain one kg body weight. The bulk density was highest for block-based ration followed by pellet, mash, and conventional rations, and the carrying capacity of truck was highest with the least transport cost in block-form of rations.

**Conclusion:**

Physical processing (mash, pellet, and block) of sorghum stover-based complete rations increased the nutrient utilization and growth performance of sheep compared to conventional chopped form. Pelleting the mash with expander-extruder procedure was found to be more profitable. Nevertheless, the cost economics revealed blocks as more preferable forms for transporting the complete rations to larger distances.

## Introduction

The deficiency of feed and fodder, both in quantitative and qualitative terms, is one of the major constraints in the sustainable development of the livestock sector. Livestock production in developing countries largely depends on fibrous feeds, mainly crop residues [1]. The nutritive value of crop residues is low and cannot meet the maintenance requirements on feeding as a sole diet. Poor palatability and low bulk density, along with low nutritive value, restrict the utilization of the crop residues as animal feeds [2]. Blending the roughage and concentrates in the form of complete feed reduces the feed wastage, feed cost, and labor cost apart from improving the nutrient use efficiency [3]. The physical form of complete feed offered as a mash, pellet, or compact feed block significantly impacts nutrient intake. The altered physical characteristics of feed may regulate ruminal fermentation process toward reduced methanogenesis with concomitant energy saving, higher metabolizable energy (ME) intake, and improved microbial protein synthesis [4]. The physical form of the ration can also affect the potential rate of consumption with pelletized rations ingested more rapidly than the mash forms [5]. Feeding the ruminants with complete feed stabilized rumen fermentation, minimized selective feeding and fermentation losses, and ensured better ammonia utilization in the rumen [6].

Pelletizing is an efficient technique in improving the intake, digestibility, feed conversion, and growth performance of livestock [7]. The heat, moisture, and mechanical pressure applied during conditioning and pelleting cause certain physical and chemical alterations having beneficial or detrimental effects on feed components, gastrointestinal development, and growth performance [8]. Besides, the application of heat and pressure during pelleting promotes starch gelatinization [9]. Pelleted meal reduces the particle size and increases the available surface for microbial degradation and substrate fermentation [10]. Further, pelleting causes a more gradual release of oil in the rumen and a higher rumen escape of dietary crude protein (CP), thereby increasing the bioavailability of dietary protein [11]. Providing complete feed in block form positively affects feed protein digestibility and energy utilization, which can be used for production while reducing the feed cost and environmental burden [4].

In India, agricultural residues are mostly burnt in the field after harvest, releasing pollutants at a large extent [12]. The low digestibility of crop residues, such as sorghum stover, is one of the main reasons for denying its usage as feed. Although the nutritive value of stover could be increased by chopping, a conventional form of feeding, its usage as a roughage source is not being encouraged in surplus fodder available areas. The favorable effects of processing crop residue-based rations against conventional chopped forms have to be explored in detail [13,14].

The study aims to determine suitable processing methods (mash, pellet, or block forms) for efficient utilization of sorghum stover-based complete rations vis-a-vis conventional feeding methods in sheep.

## Materials and Methods

### Ethical approval

Animal handling techniques and experimental protocols followed in the present study were approved by the Committee for the Purpose of Control and Supervision of Experiments on Animals (approval number SA-201715), P.V. Narsimha Rao Telangana Veterinary University, Hyderabad, India.

### Study period and location

The study was conducted from November 2016 to April 2017. The *in vivo* experiment was carried out at the college of veterinary science, P. V. Narsimha Rao Telangana Veterinary University, Hyderabad, India (17^o^12 N, 78^o^18 E, 545 m above sea level). The ambient temperature and relative humidity values during the study period were in the range of 28-42°C and 28-32%, respectively.

### Experimental rations

Experimental rations with roughage (sorghum stover) to concentrate ratio of 50:50 were formulated and the rations were processed into either mash, expander extruder pellet, or block form. The control diet comprised a conventional ration prepared by mixing chaffed sorghum stover with concentrate mixture at 50:50 proportion. The prepared rations were evaluated for performance, nutrient digestibilities, and nitrogen, calcium, and phosphorus balance in Nellore×Deccani cross ram lambs. Before starting the trial, a complete ration was processed to mash form. Then one-third of the mash was subjected to expander extruder pelleting and another one-third to compact feed block making.

### Chopping

The sorghum stover was chopped to 1.5-2.0 cm-sized pieces using a chaff cutter (Niharika Agro Mech Industries, India). The chopped stover pieces were mixed with ground concentrate ingredients at 50:50 proportion.

### Preparation of mash

The experimental feed ingredients were ground in a hammer mill (Niharika Agro Mech Industries, India) using an 8-mm sieve. The ground material, mineral mixture, and vitamin premix were mixed for 10 min in a horizontal mixer. Molasses was heated to 70°C in the preheating chamber and added into the mixer directly while mixing.

### Expander-extruder processing

The mash of complete ration was reconstituted by adding water to obtain 17-18% moisture. The material is then passed through a screw in the barrel of the expander-extruder (Niharika Agro Mech Industries, India) to attain 90-95°C by the time the pellet passes the die openings with a diameter of 16 mm. The obtained pellets were later cooled and collected into bags.

### Complete feed block making

Complete feed blocks were prepared by compressing the mash form of ration at 5000 psi (351.9 kg/cm^2^) using an electrically operated animal feed block making machine (Niharika Agro Mech Industries, India).

### Experimental animals and feeding 

Twenty-four male intact growing Nellore×Deccani cross lambs (3.5±0.5 months age, 14.50±0.41 kg) were divided into four experimental groups of six animals each in a complete randomized design. The experimental rations were randomly ­allotted to each group and evaluated for their intake, nutrient utilization, and growth performance. The experimental ram lambs were housed in individual pens (4 m×3 m) with free water access. All the animals were de-wormed and vaccinated against Peste des Petits Ruminants disease at the beginning of the experiment. The three processed sorghum stover-based complete rations were evaluated through a growth trial for 120 days and compared with the conventional control ration. The ingredient and chemical composition (g/kg) of experimental complete rations are shown in Table-1.

**Table-1:** Ingredient and chemical composition (g kg^-1^) of differently processed sorghum stover based complete rations for growth study in lambs.

Component	Control	Mash	Pellet	Block
Ingredient composition			
Sorghum stover	500	500	500	500
Maize	100	100	100	100
Cotton seed cake	80	80	80	80
Ground nut cake	40	40	40	40
De-oiled rice bran	160	160	160	160
Red gram chuni	50	50	50	50
Molasses	50	50	50	50
Urea	5	5	5	5
Mineral mixture*	10	10	10	10
Salt	5	5	5	5
Chemical composition				
OM	905	902	902	903
CP	118	119	120	120
EE	29	28	27	27
TCHO	757	755	754	756
NDF	541	539	531	530
ADF	369	367	360	362
Cellulose	262	261	255	260
Hemi cellulose	172	172	171	168
Ash	95.2	97.9	98.6	96.8
Lignin	42.3	41.5	42.1	41.0
Ca	11.2	10.8	10.9	10.7
P	6.3	6	6.3	5.9

OM=Organic matter, CP=Crude protein, EE=Ether extract, TCHO=Total carbohydrates, NDF=Neutral detergent fiber, ADF=Acid detergent fiber; Ca=Calcium, P=Phosphorus. *Mineral mixture 1 kg contains Cu-2 g, Co-100 mg, Fe-6 g, Zn-2.2 g, Ca-220g, P-100g,

Mg-40 g, Co-100 mg, Iodine-200 mg, B_1_-1300 mg, B_6_-130 mg, B_12_-3000 mg, Vit E-975 IU, Vit A-750000

IU, Vit D_3_-150000 IU

Animals were offered with experimental feeds *ad libitum* twice daily at 9:00 h and 15:00 h. Feed offered and orts, if any, were weighed daily to record feed intake. The feeding trial was carried out for 141 days duration, including the first 21 days for adaptation and subsequent 120 days for measurement of growth trial. The amount of ration was adjusted weekly as per each animal’s body weight changes to meet the nutrient requirement [15]. The experimental animals were weighed weekly before offering feed and water in the morning. Weights were recorded for two consecutive days and the body weights were represented as their mean values.

### Metabolism study

Metabolism trial was conducted after 90 days of feeding. Sheep were kept in well-ventilated individual metabolism cages (90 cm×75 cm×90 cm) to ensure feces and urine and were collected separately. Animals were allowed for a 5-day acclimatization period followed by 7 days of collection. The amounts of feed offered and refusal, feces, and urine voided per animal were weighed daily during the collection period. Feces and urine from each animal were collected daily and pooled in iron and glass containers, respectively. A representative sample of daily feed offered and refusal from individual animals was collected and pooled. After estimating dry matter (DM), the samples of all the experimental feeds, refusals, and feces were ground separately in a laboratory Willey mill through 1 mm screen and preserved in self-sealed covers. A sub-sample of feces (1/10^th^) and urine (1/5^th^) was acidified using 10% (w/w) H_2_SO_4_ to prevent nitrogen loss and then stored at −20°C to estimate nitrogen, calcium, and phosphorus.

### Feed analysis

The chemical analysis of experimental feed, residues, and feces was carried out following the methods of AOAC (2000) [16]. DM was determined by the oven drying method (934.01), CP by Kjeldahl method (VAPODEST 200, Germany) (984.13) (N×6.25), ash by muffle furnace incineration (942.05), and ether extract (EE) by Soxtherm method (SOX 406/416, Germany) (920.39). Organic matter (OM) and total carbohydrate (TCHO) were calculated by differences. The fiber components (neutral detergent fiber, [NDF]; acid detergent fiber, [ADF]) were analyzed and expressed inclusive of residual ash by the methods of Van Soest *et al*. [17]. Sodium sulfite and heat-stable a-amylase were not used in the determination of NDF. Calcium (Ca) and total inorganic phosphorus (P) in feed, feces, and urine were also estimated [16]. The density of chopped sorghum stover, concentrate mixture, and mash and pellet forms of complete rations were measured using the cubic boxes of known dimensions, while the density of block forms was calculated by measuring its weight and dimensions.

### Cost economics

The cost of feed and cost of feed/kg body weight gain were calculated by analyzing the body weight gain and total feed intake for the entire trial. Prevailing market prices of India (Hyderabad, Telangana) were taken into account to calculate the cost of individual ingredients of concentrate mixture and kg live weight.

### Statistical analysis

The results obtained were subjected to statistical analysis and tested for significance as per Duncan’s multiple range test using a general linear model. The model includes individual animals as a random effect, treatment as a fixed effect, experimental error, and initial BW as a covariate. The results were presented as means with standard errors. The means were considered significant at p<0.05 and the differences among means were determined by marking Tukey’s test. The entire statistical analysis was conducted using Statistical Package for the Social Sciences version 23.0 (IBM Corp., New York, USA).

## Results

### Chemical composition

The ingredient and chemical composition of differently processed complete rations are presented in Table-1. The differences in nutrient composition of the various physical forms were insignificant. The CP, NDF, and ADF values (g/kg DM) ranged from 118 to 120, 530 to 541, and 360 to 369, respectively.

### DM intake (DMI) and nutrient digestibility

The types of physical processing affected DMIs with the highest (p<0.05) DM consumption in the lambs fed expander-extruded pellet form of ration (Table-2). Processing of the conventional feed through mashing, pelletizing, and block making increased (p<0.05) digestibility coefficients of OM, CP, and NDF; however, no differences were observed among the different processing methods (Table-2). Further, the DM and cellulose digestibilities were tended to be higher in lambs fed processed rations. The physical processing did not affect intakes of nutrients, on per kg metabolic body weight basis, except for OM, total digestible nutrients (TDN), and ME, who tended to increase in the lambs fed mash, pellet, or block forms. The nutritive values in terms of digestible CP (DCP), TDN, and ME were higher (p<0.05) for mash, pellet, and block form of rations compared to conventional rations containing chopped stover mixed with ground concentrate ingredients.

**Table-2:** Effect of differently processed sorghum stover-based complete rations on intake and nutrient digestibility in lambs.

Parameter	Control	Mash	Pellet	Block	SEM	p-value
Nutrient digestibility (g kg^-1^)						
DM	597	613	637	613	6.6	0.07
OM	605^a^	641^b^	643^b^	631^b^	8.5	0.05
CP	642^a^	687^b^	707^b^	682^b^	8.7	0.03
EE	777	788	790	782	3.8	0.66
TCHO	570	607	607	596	9.5	0.11
NDF	559^a^	583^b^	599^b^	589^b^	5.5	0.04
ADF	506	516	532	522	6.3	0.12
Cellulose	503	512	527	515	7.3	0.06
Hemi cellulose	673	706	726	711	13.2	0.12
Nutrient intake (g d^-1^ kg^-1^ W^0.75^)						
DM	88.74	92.1	96.04	93.35	1.65	0.12
OM	80.27	83.09	86.59	84.28	1.3	0.06
DCP	6.75	7.56	8.17	7.64	0.22	0.10
TDN	49.61	54.56	57.13	54.59	1.3	0.09
ME (MJ kg^-1^ W^0.75^)	0.75	0.83	0.86	0.83	0.07	0.07
Nutritive value (g kg^-1^ DM)						
DCP	76^a^	82^b^	85^b^	82^b^	1.2	0.03
TDN	559^a^	592^b^	595^b^	584^b^	7.4	0.05
ME (MJ/kg)	8.45^a^	8.96^b^	9.01^b^	8.83^b^	0.26	0.04

^abc^values bearing different superscripts in a row differ significantly (p<0.05) (n=6). OM=Organic matter, CP=Crude protein, EE=Ether extract, TCHO=Total carbohydrates, NDF=Neutral detergent fiber, ADF=Acid detergent fiber, TDN=Total digestible nutrients, ME=Metabolizable energy, DCP=Digestible crude protein

### Nitrogen, calcium, and phosphorus balance

All the lambs of the trial were in positive nitrogen, calcium, and phosphorus balance (Table-3). Although the fecal and urinary losses of nitrogen were not affected by processing, the intake, balance, and retained nitrogen levels were higher in the lambs fed on processed rations. However, the type of processing did not affect any of the calcium and phosphorus attributes, except for urinary losses, which tended to increase (p=0.07) in processed rations. The calcium and phosphorus intake and retention were comparable among all the rations.

**Table-3:** Effect of feeding differently processed sorghum stover-based complete rations on nitrogen, calcium, and phosphorus balance (g d^-1^) in lambs.

Attribute	Control	Mash	Pellet	Block	SEM	p-value
Nitrogen						
Intake	16.92^a^	17.89^a^	19.87^b^	18.51^b^	0.83	0.05
Fecal loss	6.89	6.54	6.65	6.7	0.27	0.96
Urinary loss	5.19	5.77	6.23	5.7	0.34	0.25
Balance	4.84^a^	5.58^b^	6.99^b^	6.12^b^	0.45	0.04
Retention (% intake)	28.61^a^	31.19^b^	35.18^b^	33.17^b^	1.22	0.05
Retention (% absorbed)	41.26^a^	46.04^b^	51.25^b^	47.78^b^	1.58	0.05
Calcium						
Intake	9.99	10.13	11.22	10.27	0.45	0.34
Fecal loss	4.75	5.43	4.87	5.12	0.22	0.45
Urinary loss	1.87	1.57	2.67	1.73	0.17	0.07
Balance	3.37	3.13	3.68	3.42	0.22	0.78
Retention (% intake)	33.72	30.87	32.81	33.29	0.98	0.97
Phosphorus						
Intake	5.62	5.63	6.49	5.66	0.27	0.22
Fecal loss	1.81	1.69	1.92	1.78	0.15	0.64
Urinary loss	2.67	2.7	3.12	2.64	0.19	0.07
Balance	1.14	1.24	1.45	1.24	0.09	0.33
Retention (% intake)	20.26	21.96	22.29	21.93	1.56	0.74

^abc^values bearing different superscripts in a row differ significantly (p<0.05) (n=6)

### Growth performance

**Figure-1:**
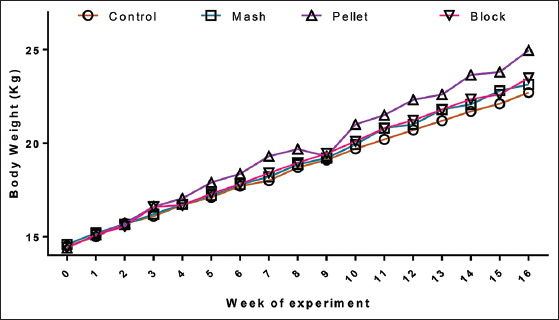
Body weights (kg) of the lambs-fed chopped, mash, pellet, and block form of rations during the growth trial.

**Figure-2:**
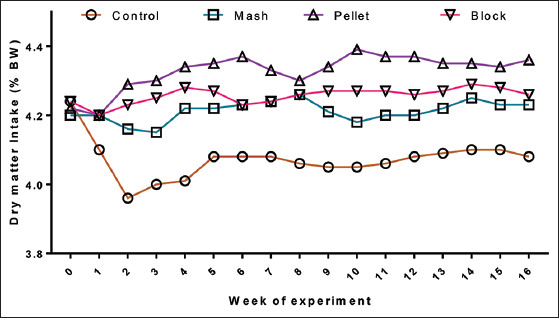
Dry matter intakes (%body weight) of the lambs-fed chopped, mash, pellet, and block form of rations during the growth trial.

Processing complete rations into different physical forms did not influence the weekly body weight changes of lambs during 120 days of feeding (Figure-1). However, the weekly DMIs were higher in pellet rations followed by block, mash, and conventional rations, respectively (Figure-2). The weight gain of ram lambs fed expander extruded pellet form of complete ration was higher (p<0.01) than conventional, mash, and block form of rations (Table-4). Feeding processed rations improved (p<0.05) the average daily gain (ADG) of lambs. Among the processed rations, pelletized rations revealed higher ADG compared to the other two processing forms. The lambs fed pelletized rations efficiently (p<0.05) utilized the feed to gain 1 kg body weight.

**Table-4:** Effect of feeding differently processed sorghum stover-based complete rations on growth rate and feed intake in lambs.

Parameter	Control	Mash	Pellet	Block	SEM	p-value
Body weight changes (kg)						
Initial BW	14.50	14.58	14.40	14.42	0.41	0.89
Final BW	22.70	23.14	24.96	23.49	0.30	0.12
Total BW gain	8.20^a^	8.56^ab^	10.56^c^	9.07^b^	0.38	0.05
Average daily gain (g d^-1^)	68.33^a^	71.33^b^	88.09^d^	75.58^c^	2.16	0.05
DM intake						
DM intake (g d^-1^)	892.6^a^	936.7^b^	1030.2^c^	960.3^b^	13.24	0.05
DM intake (g/kg^0.75^)	88.74^a^	92.10^b^	96.04^c^	93.35^b^	1.65	0.05
FCR (kg feed/kg gain)	11.07^b^	11.15^b^	9.72a	10.69^b^	0.63	0.05
Cost of feed/kg (USD)	0.179	0.180	0.183	0.181	-	-
Cost of feed/kg gain (USD)	1.98^b^	2.01^b^	1.78^a^	1.94^b^	0.11	0.05

^abc^values bearing different superscripts in a row differ significantly (p<0.05) (n=6). USD=U.S. Dollar, BW=Body weight gain, DM=Dry matter, FCR=Feed conversion ratio

### Cost economics

The cost economics analysis of feeding differently processed rations is presented in Table-5. Cost ($) of feed per kg gain was lower (p<0.05) in a pelleted ration. The cost of feed ($) per kg gain was 1.98, 2.01, 1.78, and 1.94 for conventional, mash, pellet, and block forms of rations, respectively. As anticipated, the bulk density was highest for block form of ration followed by pellet, mash, and conventional rations. Further, the carrying capacity of truck was highest with the least transport cost in block-form of rations. The combined cost of both processing and transport was highest in conventional rations than in the differently processed rations.

**Table-5:** Effect of processing on feed transport and cost economics.

Parameter	Control	Mash	Pellet	Block	SEM	p-value
Feed cost						
Cost of feed/ton (USD)	184.8	186.2	189.0	187.6	-	-
Cost of feed/kg gain (USD)	2.05^b^	2.08^b^	1.84^c^	2.01^c^	0.09	0.05
Transport cost and economics						
Bulk density (kg/m^3^)	147^a^	323^b^	442^c^	529d	26.62	0.04
Processing cost (USD/ton)	6.45	7.75	9.68	8.39	-	-
Carrying capacity/truck^a^ (tons)	6.66	14.64	20.02	23.98	-	-
Transport cost (USD/ton/						
100 km)^b^	9.73	4.43	3.24	2.70	-	-
Processing + transport cost (USD/ton/100 km)	16.63	12.71	14.27	12.36	-	-

a 1600 cubic feet carrying capacity.

b Assuming a fuel efficiency of 4 km/L and cost of diesel 0.89 USD/l. USD=U.S. Dollar

## Discussion

### Chemical composition

Processing did not affect the chemical composition of the experimental rations, presumably due to similar ingredient composition and roughage to concentrate ratio [18,19]. Likewise, few researchers reported unaltered chemical composition on processing the rations through different processing methods such as chaffing, grinding, and expander extruder pelleting [5,20]. However, Anandan *et al*. [21] reported lower (p<0.05) CP and NDF contents in block form of sweet sorghum bagasse-based complete rations compared to mash and pellets forms. In another study, Anandan *et al*. [13] reported higher ADF and acid detergent lignin values in block form of maize stover-based complete rations than mash form, despite having similar ingredients and proportions. However, the authors did not provide explanations for the alterations in the chemical composition according to the type of processing method.

### DMI, nutrient digestibility, and nutritive value

The higher DMI of pellet form of ration compared to other physical forms may be attributed to the increased palatability and acceptability of the pelleted ration. The DMI was similar in mash and block forms but higher (p<0.05) than the conventional ration. Processing decreases the particle size and collapses the cell wall structure, thereby increasing the density and hydration of feed. Compressing feed during block-making process reduces its volume and increases the bulk density [22]. The greater density will allow a faster passage rate and less rumen volume, consequently increasing the voluntary DMI [23]. Besides, the lower DMI of conventional ration might be due to the poor palatability of chaffed sweet sorghum and facilitated choice of selecting roughage and concentrate separately [3]. Similar findings on feeding bajra straw-based ration were reported elsewhere [24,25]. In a study, Anandan *et al*. [21] reported higher DMI in sheep fed mash and pellet form of sweet sorghum bagasse-based complete rations compared to those fed chops or block forms [21]. Further, Khan *et al*. [18] observed 20-29% higher DMI in lambs fed mash form than block form of complete ration. They attributed the lower DMI in block form to the temporal segregation of concentrate and roughage in block due to the larger particle size of chop form of roughage (2-3 cm), causing refusal of unpalatable portions of stover/straw. However, our study used ground form of roughage (8 mm), resulting in similar DMI of mash and block forms of rations in sheep. Higher voluntary feed intake in processed rations (mash, pellet, and block) might be explained by the avoidance of refusing unpalatable portions of roughage due to the decreased particle size. Besides, lesser retention time due to the higher rate of passage of the smaller feed particles in the mash could have favored intake to some extent at the expense of reduced digestibility [26].

The findings of this study indicate a higher intake of mash form than those fed block form of ration, leading to the higher passage rate of digesta and lower digestibility of earlier form. The digestibilities of OM, CP, and NDF were comparable between the processed rations but higher than the conventional ration. The physical form of the rations did not influence the digestibility coefficients of DM, EE, TCHO, ADF, cellulose, and hemicellulose. Likewise, Nalini Kumari *et al*. reported higher digestibilities of OM and CP in sweet sorghum bagasse-based extruded pellet form of ration than mash and chop forms in sheep [20]. Singh *et al*. [27] observed similar digestibility coefficients of DM, OM, and CP between the mash and block form of complete feed in calves. Meanwhile, Khan *et al*. [18] reported no effect of physical form on the digestibilities of nutrients, except for CP digestibility, which was lower (p<0.05) in mash form than the block form ration.

Blending, heat processing, and densification practices followed while processing might have improved the digestibilities of OM, CP, and NDF contents. Thermal processing modifies the nutritional profile through several mechanisms and provides beneficial effects on the host [28]. Heat processing causes starch gelatinization and exposes the crystalline or physically inaccessible starches (resistant starch entrapped in a cellular matrix) to enzymatic or microbial digestion [9]. The beneficial effects of pelletizing the crop residue-based complete rations on CP digestibility are well evident [29,30]. Heat processing contributes to higher microbial protein yield by increasing the ruminal fermentable starch causing efficient utilization of available nitrogen [31]. The expander-extruder processing causes a Maillard reaction, forming cross-linkages between peptide chains and carbohydrates, promoting the bypass protein into small intestine [32]. Moreover, shear force by expander would unleash the bound nutrients and increase their accessibility by rumen microbes or enzymes [33]. In the current study, the energy content of rations was sufficient for synchronization with the available nitrogen while synthesizing the microbial protein [34].

Type of physical processing (mash or pellet or block) did not affect the nutrient digestibilities. However, Anandan *et al*. [21] reported the lower OM digestibility (p<0.05) in pellet and mash form of rations as compared to the block and chop forms in sheep fed sweet sorghum bagasse-based rations. Another Iranian study found higher digestibilities of DM, CP, NDF, and ADF in sheep fed complete feed block compared to mash and pellet form of rations. These results are justified by higher (p<0.01) rumen protozoal population (total protozoa, *Entodinium* spp., and *Epidinium* spp.) in sheep fed on block form of ration compared to mash and pellet forms [5].

Processing of complete rations increased (p<0.05) the DCP, TDN, and ME contents. The DCP and TDN intakes were comparable to the values given by Dhuria *et al*. [25] in sheep fed gram straw-based complete feed in loose or block forms. The lower (p<0.01) DCP content in chop form of ration was the reflection of lower CP digestibility. In corroboration, Kumari [35] reported higher (p<0.01) DCP, TDN, and ME contents of sweet sorghum bagasse based pelleted ration compared to mash and chop form of rations fed to sheep. Reddy *et al*. [36] also observed higher DCP and TDN content of expander extruded complete ration with 40% sugarcane bagasse in buffalo bulls and Ongole bull calves. However, Singh *et al*. [27] observed that the DCP and TDN intake did not differ among calves fed either block or mash form of rations. The average daily DM, DCP, TDN, and ME intakes of lambs in the experiment were higher than the nutrient requirements at 20 kg body weight, growing at 75 g of ADG [15]. The differences in intake and digestibility of nutrients among various physical forms reflected the plane of nutrition.

### Nitrogen, calcium, and phosphorus balance

Processing increased the intake, balance, and retention of nitrogen. The higher nitrogen retention might have been attributed to the higher CP digestibility and intake of differently processed rations. Another advantage is the improved utilization of absorbed nitrogen by matching the supply of energy in the form of fermentable carbohydrates. Similarly, Reddy *et al*. [37] revealed that nitrogen balance was higher by 48% in lambs fed different fibrous agricultural residue-based complete rations processed into mash form. The differences in nitrogen retentions among the processed forms (mash, pellet, and block) are barely distinguishable. However, few authors reported higher nitrogen retention on feeding the rations processed into expander extruded pellet form compared to mash and chop forms [20,38]. Further, Khan *et al*. [18] observed higher nitrogen intake and balance in sheep fed complete rations in mash form compared to the block form.

None of the calcium and phosphorus balance-related parameters was affected by processing the rations. The calcium and phosphorus balance in herbivorous animals could be directly related to their levels in feed consumed [39]. Although not significant, a trend of higher urinary losses of calcium and phosphorus was observed in pelleted rations, probably due to the higher levels of the respective minerals in pelleted form of ration [40]. Similar to present findings, no significant effect on calcium retention was observed in calves and buffalo bulls fed sugarcane bagasse-based expander extruded pellet compared to conventional ration [41,42].

### Growth performance

The weight gain and ADG were higher in pellet-fed groups compared to conventional, mash, and block form of rations. Our findings appear to be substantiated by Reddy *et al*. [43] and Anandan *et al*. [21], who reported that pelleting improved daily weight gain in lambs and kids compared to chop, mash, or block form of rations. The higher weight gain and ADG in pelleted ration are reflected by the increased digestibilities of OM, CP, and NDF due to the synchronization of available energy with nitrogen. Similarly, Kumari *et al*. [20] reported higher weight gain and ADG of lambs fed sweet sorghum bagasse based complete rations processed in expander extruded pellet form compared to those fed mashes or chop forms.

Expander-extruder pelleting of mash improved ADG on feeding sugarcane bagasse-based complete ration to calves [42]. Pelletizing the ration increases the simultaneous intake of roughage and concentrate, optimizing the rumen environment, consequently increasing the performance compared to mash, block, and chopped forms. However, the effect of type of processing on weight gain and ADG was not constant throughout the literature, and few researchers reported several inconstancies. For instance, Yasir *et al*. [44] reported that the lambs fed on oat straw-based complete feed recorded higher ADG in block-fed group than mash fed lambs. Higher ADG was observed in sheep fed mash form of maize stover based complete ration compared to block form [13]. Further, Singh *et al*. [27] observed similar weight gain and ADG in the calves fed wheat straw-based complete feed in the form of blocks or mash.

Higher nutrient digestibility and efficient utilization of absorbed nutrients in pellet form of ration might have resulted in better feed conversion ratio (FCR) in lambs fed pelleted ration. The beneficial effects of pelletized rations over mash or chopped forms are well evident [38]. The poor feed efficiency of chopped ration might be attributed to lower DMI because of the feed particles’ segregation due to differences in bulk density of roughage and concentrate. In contrast to our findings, Karimizadeh *et al*. [5] showed that feeding the complete feed blocks improved FCR compared with the mash and pellet rations in lambs. Further, Singh *et al*. [27] observed similar FCR in crossbred calves fed with wheat or rice-based complete ration in either mash or block forms.

### Cost economics

Expander extrusion required additional power consumption for steam production, and hence the cost of processing the pelletized ration increased by 2.92% over the mash ration. Feeding expander-extruded sorghum stover-based complete ration reduced the cost of feed per unit live weight gain by 11.87%, 13.83%, and 9.85% in comparison to conventional, mash, and block forms, respectively. Despite the higher processing cost, the cost of feed per kg gain was lower in pelleted ration compared to other rations because of the increased efficiency of nutrient utilization and higher weight gains. In accordance with our results, Kumari [35] observed that feeding expander extruded sweet sorghum bagasse-based complete ration was more economical compared to chop and mash forms in sheep.

Bulk density of feed, defined as ratio of the bulk mass to volume of its fit, plays a crucial role in determining the handling, storage, or transport costs. Because of high fuel and labor prices, high bulk density of feed decreases the cost of handling and transportation [13]. This notion is more pertinent for transporting low-dense rations such as roughages having high bulkiness. The cost of transporting one-ton feed to 100 km distance through a truck having fuel efficiency of 4 km/l was least in block form (2.70 $), followed by pellet (3.24 $), mash (4.43 $), and chopped (9.73 $) forms. A major drawback for pellet and block forms of rations is their higher processing costs compared to chopped and mash forms. However, these costs were covered by their higher carrying capacity, lower transportation cost, and better feeding value. Summation of processing and transport cost revealed block as a more economical processing form while considering the off-farm expenditure. Although the growth trial revealed higher weight gains in pelletized rations, the overall profit depends on the total distance of the ration to be transported before reaching the farm. In case of farther distances, transporting the block forms would be more advantageous than carrying pelletized rations.

## Conclusion

The study concluded that different physical processing forms (mash, pellet, and block) are more advantageous in terms of nutrient utilization and growth performance of sheep compared to conventional chopped form of sorghum stover-based complete rations. Although the mash, pellet, or block forms have enabled effective utilization of sorghum stover up to 50% level in complete rations, pelleting the mash with expander extruder procedure was found to be more profitable due to the higher body weight gains, better FCR, and lower feed cost per kg gain. However, the cost economics revealed blocks as more preferable forms for transporting the complete rations to larger distances.

## Authors’ Contributions

JN, NNK, TR, and VCP: Designed the study and interpreted the results. JR: Conducted the trial and laboratory analysis. AAK: Supervised the analysis. JR and PRKR: Interpreted the results and drafted the manuscript. All authors read and approved the final manuscript.
